# Comparing Efficiency and Performance of IoT BLE and RFID-Based Systems for Achieving Contract Tracing to Monitor Infection Spread among Hospital and Office Staff

**DOI:** 10.3390/s23031397

**Published:** 2023-01-26

**Authors:** Maggie Ezzat Gaber Gendy, Phi Tham, Flynn Harrison, Mehmet Rasit Yuce

**Affiliations:** Department of Electrical and Computer Systems Engineering, Monash University, Melbourne, VIC 3800, Australia

**Keywords:** COVID-19 pandemic, contact tracing, hospital/office settings, BLE, RFID, indoor infection spread, human–human proximity, IoT, wireless sensing systems

## Abstract

COVID-19 is highly contagious and spreads rapidly; it can be transmitted through coughing or contact with virus-contaminated hands, surfaces, or objects. The virus spreads faster indoors and in crowded places; therefore, there is a huge demand for contact tracing applications in indoor environments, such as hospitals and offices, in order to measure personnel proximity while placing as little load on them as possible. Contact tracing is a vital step in controlling and restricting pandemic spread; however, traditional contact tracing is time-consuming, exhausting, and ineffective. As a result, more research and application of smart digital contact tracing is necessary. As the Internet of Things (IoT) and wearable sensor device studies have grown in popularity, this work has been based on the practicality and successful implementation of Bluetooth low energy (BLE) and radio frequency identification (RFID) IoT based wireless systems for achieving contact tracing. Our study presents autonomous, low-cost, long-battery-life wireless sensing systems for contact tracing applications in hospital/office environments; these systems are developed with off-the-shelf components and do not rely on end user participation in order to prevent any inconvenience. Performance evaluation of the two implemented systems is carried out under various real practical settings and scenarios; these two implemented centralised IoT contact tracing devices were tested and compared demonstrating their efficiency results.

## 1. Introduction

Starting in 2020, the world has been witnessing the COVID-19 pandemic that has affected people’s economic [1], physical [2], psychological [3], mental [4], and social [5] life aspects [6]. Many countries have taken rigorous measures: cities began locking down, international travelling was completely banned, and there was an urgent direction and tendency to contain the virus using currently available technologies. One efficient method of containing the virus is through contact tracing [7], which is—as defined by the World Health Organization (WHO)—the procedure for identifying and monitoring people who have been exposed to an infected case [8] so they can isolate and further reduce the spread.

Contact tracing has been effectively adopted to contain historical outbreaks of Ebola, human immunodeficiency virus (HIV), and measles [9]. However, at that time, the traditional contact tracing was done manually, which was extremely time-consuming, labour intensive, inefficient, highly prone to errors, and not scalable [10]. In response to that, digital contact tracing, a more autonomous method, was deployed during the early stages of the COVID-19 pandemic [11]. Many countries, such as Singapore [12], Australia [13], China [14], and India [15], adopted a range of new contact tracing technologies [10], such as Wi-Fi [16], Bluetooth [17], cell tower triangulation [18], Global Positioning System (GPS) [19], QR codes [20], Zigbee [21], RFID [9], along with IoT [22].

IoT is an emerging concept that allows smart communication between multiple electronic devices and sensors through the Internet without requiring human interaction [23]. Common IoT applications are for smart homes [24], smart farming [25], smart transport [26], smart cities [27], and smart security [28]. The architecture of IoT consists generally of four main layers [29,30]. The first is the perception layer for dealing with all the electronic devices and sensors. The next is the transport layer, which allows physical devices to communicate with the network in various ways, such as using the TCP/IP stack, via gateways or wirelessly through Wi-Fi/3G/4G. The third layer is the processing layer for processing the incoming data. The final layer is the application layer that helps visualize the processed data to help answer business questions.

Using IoT in contact tracing can enhance its scalability and automation and can handle the ever-increasing number of contact tracing tasks [31]. Generally, there are two common contact tracing architectures: centralized and decentralized [32]. In a centralized architecture, users share their anonymous assigned IDs with a central server where contact tracing and risk analysis are done. In decentralized architecture, however, the users download the database from the server and perform the risk analysis on their devices [33].

RFID and BLE are among the most common wireless sensing technologies used in contact tracing. Developments in RFID chip production are making it viable for new applications and contexts [34]. Usually, an RFID system consists of a tag, a reader, and middleware. Based on the operating frequency (low frequency, high frequency, ultra-high frequency, microwave, or ultra-wide band), the system’s performance, operating range, and power requirements will differ [35]. Despite the advancements of RFID technology, some technical issues still appear due to environmental factors; for example, noise may interfere with the reader, causing it to miss some tags, which negatively affects the read rate (It represents the number of tags that can be scanned in a specific amount of time whether it is the same tag or multiple tags.) [34]. In addition, the prices for RFID systems remain relatively expensive compared to other available technologies [34]. Regarding the healthcare industry, the adoption of RFID technology is relatively recent compared to other fields such as education (libraries), retail, supply chain production, and logistics [36].

Bluetooth has also been used by many countries designing automated contact tracing [37]. Bluetooth low energy (BLE) is a low power standard introduced to the Bluetooth 4.0 standard in 2009 that uses the unlicensed 2.4 GHz band for communication [10]. BLE only sends short messages either as broadcasting advertisements or data packets between the transmitter and receiver. These packets are sent on three advertising channels (37, 38, 39) in order to not interfere with standard Wi-Fi channels, which makes it suitable for indoor environments [38]. Technically, the device transmits broadcast packets to these three channels throughout each of its advertisement spans. The scanning mode is used by recipients to listen for such advertisement packets. During each scanning window, they record communications, either actively or passively. The former allows the scanning gadget to request further information from the advertiser. In contrast, gadgets in passive mode do not communicate with one another; instead, they only collect information from broadcast messages. A common method for performing localization with BLE beacons is to use the receiver signal strength indicator (RSSI) to measure the distance between the receiver and transmitter with a propagation model [9]. This method can also be done with multiple beacons to perform triangulation obtaining a relative location as well as used for room level localization [39,40]. Another typical solution is to take advantage of the smartphone Bluetooth radio, which is a ubiquitous feature, by having the population install an application on their smart phones. These applications will then periodically send out BLE advertisements that contain some information about the user,. When another phone detects the packet, it will determine the distance from the first device using the RSSI. After this exchange, the close contacts get notified depending on the applications architecture as previously noted [9]. Examples of some countries and their apps are Brazil (SUS) [41], Vietnam (Blue zone), Singapore (TraceTogether), South Korea (Corona 100 m), Australia (COVIDSafe), India (Aarogya Setu), and USA (Safe Paths) [42]. However, many researchers have pointed out for the impracticability for hospital and offices staff to continuously carry their mobile devices and, more importantly, these devices could be potential carriers for infection within indoor environments [43,44].

The success of any system must follow three critical success factors levels as defined in [45]. The first is the strategic level, which involves developing a clear strategy; the second is the tactical/management level, which includes integrating the system infrastructure and proper staff usage. Finally, there is the operational level, which addresses avoiding major process changes, integrating the data collected, and using cost-effectiveness components.

This research presents autonomous, low-cost, long-battery-life IoT solutions for contact tracing that follow the three success factors levels. These solutions function without an end user dependency in hospital/office setting environments to protect the staff from indoor virus transmission. This is done by measuring the proximity between staff and tracking their presence as well as duration in commonly used indoor areas. This was based on the practicality and success of Bluetooth and RFID radio frequencies used in IoT devices for localization. Through this study, two implemented proposed contact tracing devices are described, compared, and tested, starting from concepts through final product examination.

The article is divided into seven sections. Section 2 reviews the related work for BLE and RFID technologies in contact tracing. Section 3 thoroughly describes the BLE-based system, Section 4 explains the RFID-base systems, and Section 5 displays the tests and results. Finally, our work is discussed in Section 6 and concluded in Section 7.

## 2. Related Work

Regarding using RFID and Bluetooth in automated contact tracing for pandemic outbreak management, some trials were conducted employing each technology. For tracking the severe acute respiratory syndrome (SARS) pandemic, Singapore deployed an RFID system created by Singapore’s Department of Defence, Science, and Technology in collaboration with ST Engineering [46]. In Taiwan [47], the Industrial Technology Research Institute built another system. These two systems shared numerous commonalities: both systems were used in hospitals and employed active RFID tags that communicated with readers through ultra high frequency (UHF) bands. The only difference between the two is who was tagged: RFID tags were provided to all hospital employees, patients, and visitors in Singapore; however, only medical personnel in Taiwan were issued RFID tags. These systems showed major challenges, including integrating the data collected and the costs of the installed systems. Another trial used ultra high frequency for accurate indoor localization [48]. This system introduced a new concept of semi-passive RFID tags; it can sense the location of this semi-passive tag using the backscatter signals from normal passive RFID tags. The drawback of this system is its practicality; it needs a large number of fixed passive tags to detect one semi-passive tag. A trial in proposed an IoT RFID based automated tracing and tracking method, which used RFID tags and the near field communication (NFC) protocol-based mobile application as a reader. This study asked people to install a specific application on their smartphones and leave it open in the background, which made the study dependent on human interaction. Another study [49] used the RFID together with GPS in a wearable contact tracing wristband; however, many people raised privacy concerns about using GPS in contact tracing.

On the other hand, many countries and trials used Bluetooth for contact tracing. Some approaches for positioning and proximity detection (especially indoors) were presented in [50,51,52,53,54,55,56]. These works use the RSSI to measure distance between receiver and transmitter. One study [57] demonstrated that Bluetooth 2.0 can be utilised for localisation with an inaccuracy of less than 45 cm. However, Bluetooth has the disadvantage of being an active protocol that requires two parties to establish a connection before any payload can be sent. Because of the extra complexity of the connection formation, this may impede effective message exchange. Furthermore, because devices advertise themselves, they alert potential attackers to the location of an engaged interface.

In the same manner that distance measurements for conventional Bluetooth are used, RSSI is used to determine the distance between transmitter and receiver in BLE. Various signal propagation models could be utilised to achieve this goal (e.g., the exponential or the polynomial model). BLE is more suitable than Bluetooth for co-location detection and distance measurements due to its passive advertising/broadcasting and lower energy consumption. As a result, BLE became widely employed as a contact tracing technology as developed in [58,59,60]. However, these studies used people’s personal devices, such as smartphones, smart watches, or fitness trackers, which are distinctively different in their transmission power as well as their antennas gain/radiation patterns. RSSI readings must be calibrated to the appropriate instruments. Moreover, some wearables were introduced for the sake of contact tracing, such as Easy band [61] and Abeeway [62], which used BLE and Wi-Fi. These wearables give optical, acoustical, or mechanical alerts to maintain social distancing. These wearables have some limitations, such as limited accessibility and the trade-off between user comfort and device complexity.

BLE has also some limitations, including that distance measurements can be inaccurate due to multi-path and shadowing effects produced by signal reflection, walls, and objects that obstruct wireless signal propagation. Furthermore, an advertisement packet’s payload is limited to 31 bytes. Approaches that use passive scanning can save significant amounts of energy as compared to systems that use active message exchanges. BLE, like Bluetooth, contains vulnerabilities that allow attackers to exploit it when it is turned on. Moreover, this method is bound to produce false-positives and false-negatives, which affect the system performance and efficiency [42]. The authors in [63] were able to correctly detect 100% of risky contacts in 15 min at a distance of 2 m while accepting a 30% false-positive rate.

In this study, we offer two fully constructed IoT wireless sensor systems for contact tracing and monitoring infection spread. Most trials in literature sacrificed one aspect over another, such as power consumption, cost, simplicity, performance, or design. We claim that we solved the previously mentioned problems without sacrificing any aspects, based on our results, design, and hardware. First, our systems do not rely on people’s smart devices or wearables, which avoids concerns such as virus transmission carried by these devices, privacy violations, impracticability, technical variations between devices, and power consumption payload off individuals’ personal gadgets [61,62]. In addition, our systems are completely independent from human interaction; no actions such as installing applications, granting them permissions [9,41,42], code scanning [20], or tag taping [64] are required; to improve simplicity and performance, our system will not be prone to human errors. Furthermore, in the implementation of our RFID system, we give special care to guarantee that the beacon is in the best location to minimize any partial gaps in contact tracing that might result in missing personnel interaction, as noted in some former studies [65,66,67]. Although some studies have shown that RFID tags are unable to directly convey the extent of an exposure, we used the RSSI values in our system to overcome this limitation. Moreover, unlike some RFID systems discussed in the literature [48], our RFID system does not require large numbers of tags to identify the individuals; each individual needs only one tag to be sensed, which reduces expenses and system complexity. Additionally, our systems are cost effective as compared to some trials, such as the BLE application COVIDSafe, which had predicted development and operational expenditures of AUD 6.75 million through early 2021, with additional monthly maintenance costs of around AUD 100,000 [13]. In contrast, our systems used low-cost and power efficient off-the-shelf sensors. In relation to privacy preserving purposes, our system is able to successfully track hospital/office staff inside the premises only where beacons/readers are installed, as opposed to other systems using geo-localisation [68,69].

In the following sections, we describe the comprehensive system architecture of the two proposed autonomous IoT contact tracing platforms, discussing briefly the hardware, software, and web interfaces of each system.

## 3. BLE Based Contact Tracing System

**Figure 1:**
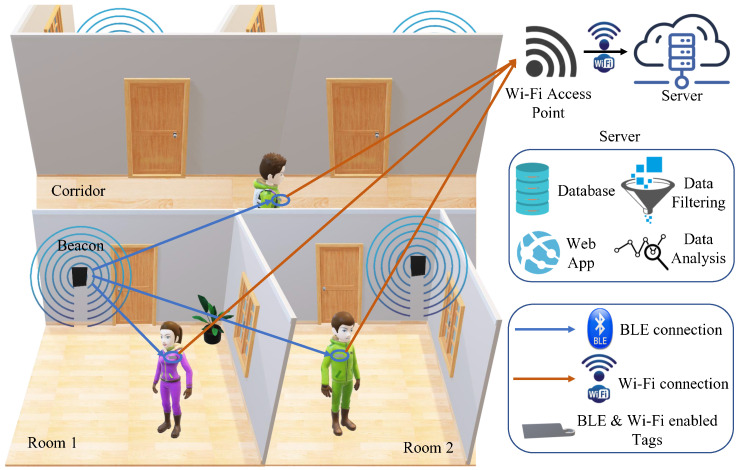
BLE based contact tracing system architecture overview.

Because of the low cost presented by BLE indoor localization methods and the standard availability of Wi-Fi in modern buildings, this study makes use of these two technologies to detect close contacts and measure staff proximity for indoor environments. The comprehensive system architecture is shown in Figure 1. It consists of BLE beacons, BLE and Wi-fi enabled tags, and a server. The beacons are low power devices that are used to periodically broadcast specific identifying data to all listening devices. They are implemented in each room of the office or the hospital to distinctively identify the room. The data transmitted between the beacons and the tags are passively transmitted, with no need for connecting, pairing, or bonding with the devices (i.e., no peer-to-peer connection needed). The tags are responsible for listening to the beacons’ advertisements, recording the beacons’ RSSI received values, as well as transmitting the data to the server over Wi-Fi upon connecting to a Wi-Fi access point.

The system calculates threshold RSSI values in advance using propagation models (as will be further discussed in coming sections) to define different levels of proximity to the beacons. Comparing the received RSSI value of this room’s beacon against the predetermined RSSI threshold values, the server back-end decides whether a tag is within a room (entry and exit detection). When a tag enters a room, the RSSI value increases above the threshold. Close contacts can be determined when two tags enter the same room at the same time interval.

### 3.1. Hardware

**Figure 2:**
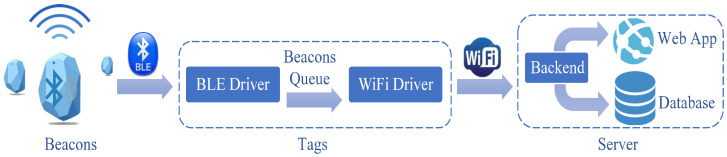
BLE contact tracing IoT system architecture overview.

The system hardware consists of two main components: beacons and BLE/Wi-Fi equipped tags as shown in Figure 2.

Beacons: The choice of the Bluetooth beacons controller was heavily driven by a months-long battery management requirement. Several trials were made using ESP32-C3 and the old original ESP32 microcontrollers, but upon testing they were found to be nonviable due to continuous high current draw. A more power-efficient system on a chip (SoC) was the nRF52810, which has a significantly lower current draw at both BLE transmission powers of 0 dBm and +4 dBm of around 4.6 mA and 8 mA, respectively [70]. It is a cost-effective and high-performance Bluetooth 5.3, 2.4 GHz transceiver SoC. Its microprocessor is 64 MHz, 32-bit Arm Cortex-M4, and it has a flash memory of 192 KB and RAM memory of 24 KB. In addition, nRF52810 has both a low drop out linear regulator and a high efficiency DC/DC switch mode regulator, which does not require other integrated circuits (ICs) in the beacon design.

**Figure 3:**
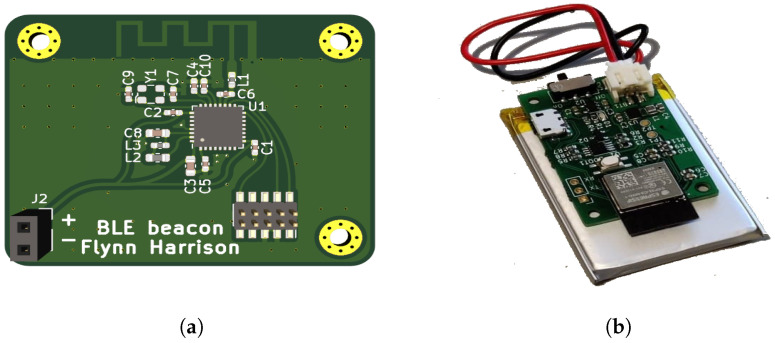
BLE contact tracing IoT system hardware: (**a**) the top side of the BLE beacon, (**b**) the BLE tag.

For the beacon antenna, a printed circuit board (PCB) design was chosen compared to a chip antenna for lower cost. The antenna chosen is a meandered inverted F design, chosen from a list of reference designs produced by Texas Instruments [71], for its popularity in Bluetooth and other 2.4 GHz embedded devices. The final beacon design is shown in Figure 3a.

The microcontroller is fed by a CR2450 battery that has a 550 mAh capacity. The estimated power usage was calculated using the manufacturers power profiling tools ‘’Online Power Profiler for BLE” [72]. It estimated a total average current of 14 μA (11.36 μA for BLE events, 1 μA for LF clock calibration current, and 1.2 μA Idle current). Depending on these figures, the battery capacity estimate is 1636 days.

Tags: The tag is quite important in this system, as it performs the majority of the work. The ESP32 C3 single-core Wi-Fi and Bluetooth 5 microcontroller SoC was chosen over the other versions (the single RISC-V core and the old Xtensa dual core design) due to the better power efficiency. A rechargeable lithium-polymer (LiPo) battery of 1100 mAh was selected to power the tag, as shown in Figure 3b. The charged battery time was measured to be 16 h and 18 min, which is the time between the tag’s first transmitted RSSI measurement to the database and the last one before it runs out of battery (more specifically when the battery goes below 3.446 V, where an under-voltage protection circuit turned off the device).

**Figure 4:**
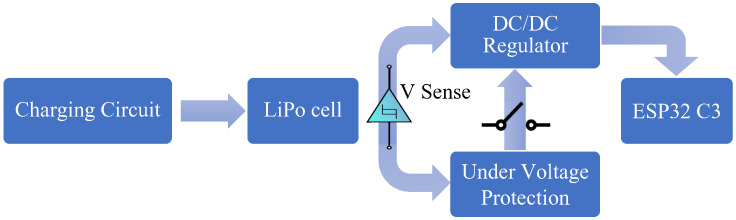
The BLE tag hardware circuit overview.

The implemented hardware overview of the tag can be seen in Figure 4. The tag contains a battery charging IC for charging the LiPo at 500 mA rate. A RP515x DC/DC buck converter (regulator) is used, because the LiPo has a variable output voltage. The regulator is capable of providing 300 mA needed by the ESP32, and it has an efficiency of approximately 95% when voltage is between 3.6 and 4.2 Volts [73]. For protection from over discharge, an analogue under-voltage protection circuit was designed and implemented. This circuit used a Schmitt trigger to turn off the DC/DC buck converter once the voltage drops below 3.4 V with a 100 mV hysteresis loop to deal with the battery voltage rising when there is no longer under-load. A switch is placed between the protection circuit and the buck converter to allow for manual turn off. The prices for beacon and tags materials used for the BLE system are shown in Table 1.

### 3.2. Software

As previously noted, the system consists of BLE beacons, tags, and a server. The beacons periodically rather than constantly advertise their data in order to conserve power, and the advertising interval was chosen to be 1000 ms [74]. Between the advertising intervals, the device is in its light sleep state that only uses 1.5 μA. This also helps in reducing the interference between other Bluetooth devices that would affect the RSSI readings. The advertising data come in the form of packets including manufacturer data bytes and a 32-bit unique identifier (UID) for beacons identification, as seen in Table 2.

**Figure 5:**
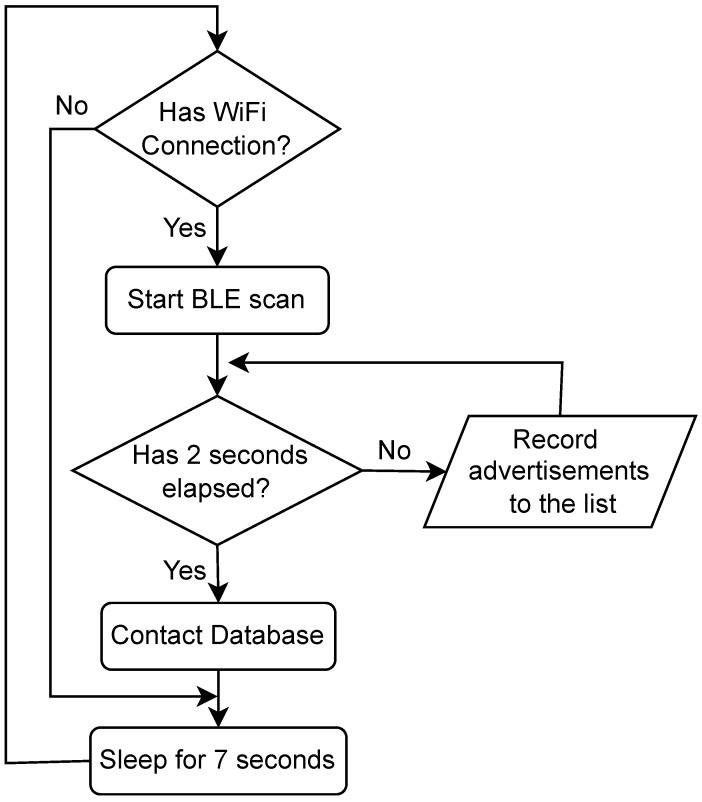
Operation flowcharts for BLE wearable tag.

The operational flowchart of the BLE wearable tag is shown in Figure 5. All tasks are performed in a single loop that has a period of 10 s; although this loop could be faster to improve the localization, the power consumption due to radio usage would have been heightened, resulting in lower tag battery life. On startup, the tags attempt to connect to an indoor Wi-Fi access point; this is pre-configured in the software to be either a Wi-Fi Protected Access 2 (WPA 2) enterprise or WPA 2 personal. If the tag loses connection or does not find a network, it will keep scanning for a known service set identifier (SSID). Then, it listens for any advertising events for 2 s to avoid missing any of them due to interference. The list of recorded beacons with their RSSIs are then packaged with a packet ID that iterates with each packet in order to keep track of the beacons heard at approximately the same times with different tags. This appended list is then transmitted to the server via use of a series of HTTP post requests that contain the packet ID, beacon UIDs, and the measured RSSIs. The tag then goes into a lite-power saving mode when the Wi-Fi radio is not being used for transmission, which enhances the battery life.

The received values of the beacons’ RSSI are used for contact tracing and human proximity calculations. These values are dependent on the distance from tags and broadcasting power value: the BLE broadcasting power value is around 2–4 dBm—and therefore, the RSSI value will be around −26 (a few inches) to −100 (40–50 m distance). The distance *d* between beacon and tag can be measured according to the following equation:

(1) $$RSSI_{dBm}=RSSI_{do}-10\times n\times log_{10}\left(\frac{d}{do}\right),$$

where *n* is the propagation constant or path-loss exponent, which is selected as 2.5 for a standard office environment [34]; do=1 m, and $RSSI_{do}$ is the average measured received RSSI at 1 m that was found to be −57 dBm [59]. This equation is used to find threshold values for determining different distance ranges. Determining in what room a tag is located through calculations is done with some assumptions, primarily that the beacons are placed centrally within the room and that the walls of the room have a significant effect on the RSSI reduction.

**Figure 6:**
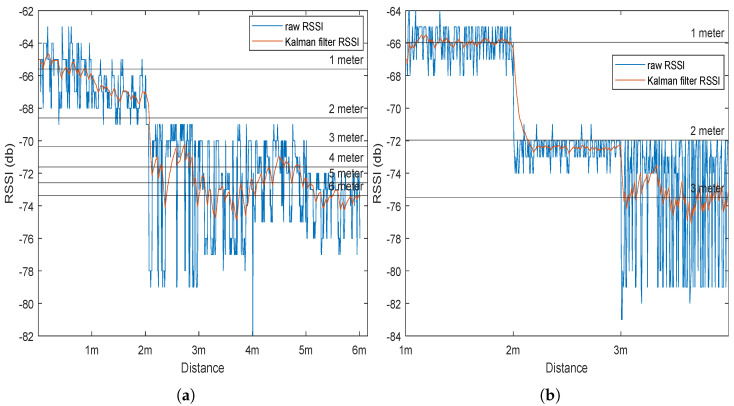
RSSI measured values at 1 m intervals against the predetermined threshold values vs, distance in an (**a**) outdoor environment, (**b**) indoor environment.

To test the validity of the threshold values obtained, two tests were conducted. The first was outdoors with *n* (multi-path factor) being lowered to 1, and the second was conducted indoors. The tests involved placing a tag and a beacon on stands at the same height, then, for each distance interval of 1 m, an average of 150 samples of RSSI data were recorded. These data are then plotted and compared to the expected calculated threshold RSSI values. From Figure 6, it can be seen that there is a general decreasing trend for the RSSI with increasing distance. In Figure 6a, distances were examined reaching 6 m, and a large amount of error was observed due to outdoor interference and multipaths. Figure 6b shows the results of the indoor environment with smaller distance tested, where the results came far closer to matching the predicted RSSI values. The mean indoor error for 1 m was 0.0076, for 2 m was 0.99, and for 3 m was 1.99 dB for filtered RSSI.

As the RSSI values tend to fluctuate, at the server the received data from the tags are filtered by a Kalman filter and stored upon upload. The Kalman filter is an iterative state predictor that uses the history of noisy observations, and it has two stages: forecasting and updating. First, the filter defines the current RSSI state $x_{t}$ as a combination of the former state $x_{t-1}$ and noise ϵ (the system process noise) [75,76,77]. Then, it defines the observation model to get the observation measurement $z_{t}$:

(2) $$z_{t}=x_{t}+\delta_{t}$$

where δ is the measurement noise caused by faulty measurements.

The forecast step: This step describes the expectation of the state without using any measurements.

(3) $$\bar{\mu_{t}}=\mu_{t-1}$$

(4) $$\bar{\Sigma_{t}}=\Sigma_{t-1}+R_{t}$$

The forecast is described by μ, the bar above the prediction indicates that it still needs to integrate knowledge from the measurement in the update stage, and Σ determines the certainty of our prediction; R defines system noise; we select a low value for process noise (R = 0.01), believing that the majority of the noise is created by measurements. Using these predictions, Kalman gain can be computed:

(5) $$K_{t}=\bar{\Sigma_{t}}{\left(\bar{\Sigma_{t}}+Q_{t}\right)}^{-1}$$

The gain is used to weight the certainty of our estimate versus the certainty of the measurement (influenced by the measurement noise Q); Q is given a value that corresponds to the noise in the measurements (the variance of the RSSI signal). The variance of the signal strength in this study was determined by taking the variance of a sample set taken at 1 m from a beacon: it was found to be 1.0682. This translates directly to the update step:

(6) $$\mu_{t}=\bar{\mu_{t}}+K_{t}\left(z_{t}-\bar{\mu_{t}}\right)$$

(7) $$\Sigma_{t}=\bar{\Sigma_{t}}-\left(K_{t}\bar{\Sigma_{t}}\right)$$

**Figure 7:**
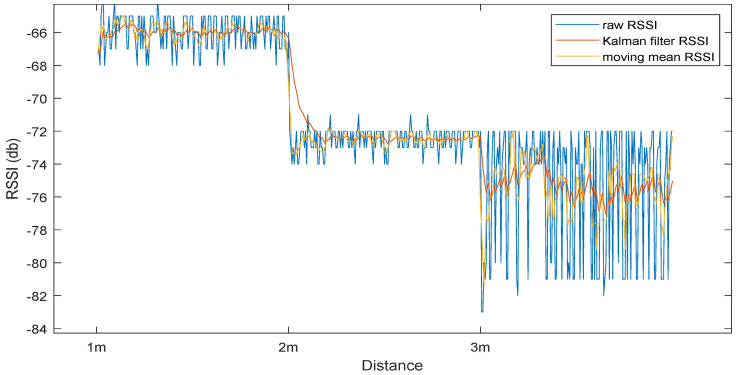
Received signal strength indicator (RSSI) raw data compared to Kalman and moving average filter RSSI.

In the update stage, the final prediction of the system (μ without the bar) and the certainty Σ are calculated. To test the effectiveness of Kalman filter, it is compared against a moving mean filter with a window size of 5. From Figure 7, the Kalman filter showed better results than the moving average filter. At the 3 m measurements, the Kalman filter has a standard deviation of 1.51, whereas the moving mean had a higher standard deviation of 1.72. A limitation of the Kalman filter that can be seen in the 1 m to 2 m step is that its response time is rather slow.

**Figure 8:**
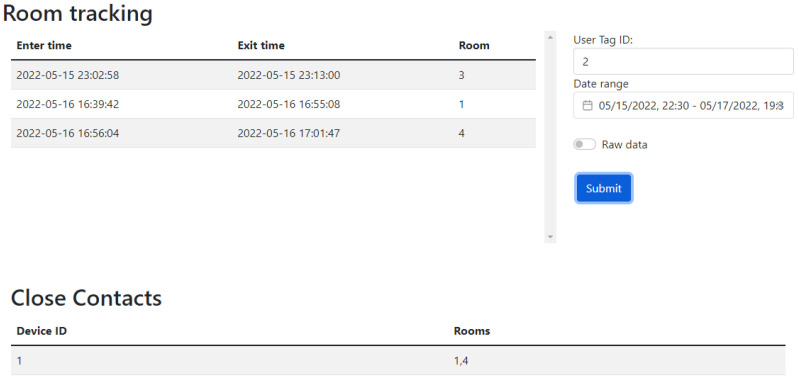
BLE system web interface displaying the information found on the database for tag 2 in the displayed time range. This information includes the rooms enterance and exit times as well as the close contacts that were in the same rooms at the same time.

The server in this study has three components: the web application front-end for user interfaces and results monitoring, the back-end for data handling, and a MySQL database for data storage. The front-end is built on Vue 3 to allow the web page to have reactive elements, and the back-end uses a Python-based server called Flask to keep the development time down for the data processing. The web interface (front-end) has three main divisions, two tables (for room tracking and close contacts) and an input panel, as shown in Figure 8. The input panel allows the user to look up a certain tag ID and specify a time frame of interest. These data are then passed to the back-end to retrieve and format the data, where they can be handed back to the front-end. One of the tables shows a list of all the rooms that a tag has visited with time stamps of the entry and exit times, and the other table lists all the other tags that have been in the same room at the same time as the tag ID that is being searched.

To reduce the chance of the database being abused, the database has users set up with permissions, which only give access to permitted individuals to use SELECT and INSERT commands on the data stored.

## 4. RFID Based Contact Tracing System

**Figure 9:**
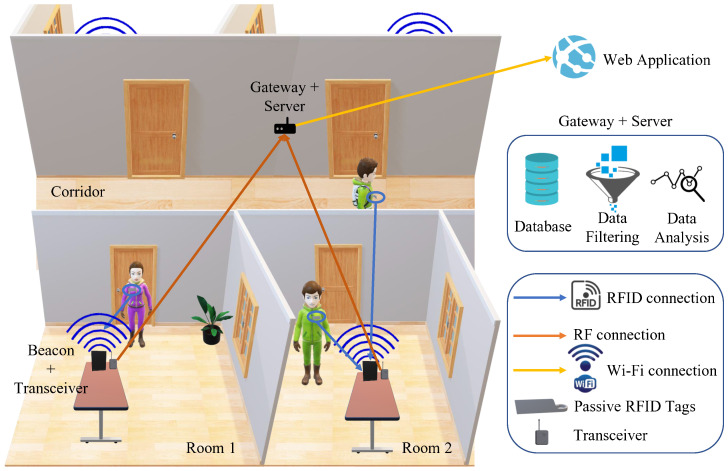
RFID based contact tracing system architecture overview.

This study investigates the use of high frequency (HF) and ultra high frequency RFID systems (UHF) in a low-cost contact tracing application. The overall system overview can be seen in Figure 9. It consists of an RFID reader, tags, and a gateway. It captures details such as name and contact details from the tags; it also tracks the rooms the tags have been in and gives indications of human-to-human proximity.

**Figure 10:**
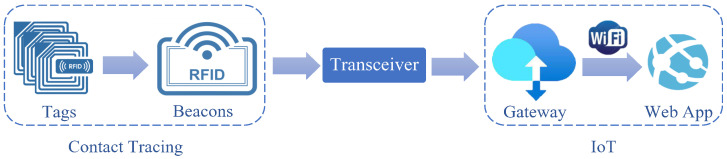
RFID contact tracing IoT system overiew.

The reader/beacon usually consists of a transmitter and a receiver, where the transmitter broadcasts a signal searching for any tags within its range. Once an RFID tag has received this signal from the reader, the tag back-scatters the received signal [78], sending its own signal back to the reader. The reader will then capture the back-scattered signals by its receiver, so it can locate the tag; following that, the transceiver transfers the data to the gateway. The proposed system can be divided into two parts as seen in Figure 10. The first part is responsible for contact tracing and is done by the RFID tags and the reader along with the microcontroller unit, and the second part is the IoT part, including the gateway that hosts the database and is connected to the web app. The transceiver is the communication between the contact tracing and the IoT component of the project.

### 4.1. Hardware

The system hardware consists of beacons, tags, transceivers, and the gateway. Two RFID beacons were implemented (one for each frequency band); however, the transceiver modules and gateway were shared between the HF and UHF systems as the performance for these two components does not differ between the systems. Beacons:

- HF RFID: The microcontroller chosen for the HF RFID system was the ATmega328P Arduino Uno board; it was chosen over other microcontrollers, such as ARM cortex-M7, due to its wide availability, relatively cheap price, and available open-sourced libraries [79]. The HF RFID reader used was the RC522 RFID reader, as it is relatively cheap, it has a MFRC522 chip onboard for the reading/writing work, and it also comes with an inbuilt antenna that operates at 13.56 MHz. Its operating voltage is 3.3 V and it consumes 30 mA. The communication between the RC522 and the Arduino board is done via full duplex serial peripheral interface (SPI) communication protocol, as both the master (Arduino) and slave (RC522) transmit data simultaneously [79].
- UHF RFID: The microcontroller used was the ATmega2560 Arduino Mega board, it regulates the input voltage down to 5 V using LMV358LIST STMicroelectronics Operational Amplifiers and SPX1117M3-L-5-0/TR – Linear Voltage Regulator IC. The Z6334 DC/DC buck converter with output 5 V and S9V11F3S5 (3.3 V–1.5 A) step down voltage regulator were used with the design. The M6E Nano RFID reader was picked to be the UHF RFID reader for its good price to performance ratio; it operates at frequencies ranging from 859 to 920 MHz and at minimum output power of 5 dBm and a maximum of 27 dBm. For this study, the 920 MHz frequency and 20 dBm output power were used. Although the M6E nano reader comes with an inbuilt antenna, the performance was not up to standards, and the reading distance was only up to 30 cm. For this reason, an external antenna was used instead, operating at a similar frequency range as the reader and at a maximum power of 100 W with a gain of 6 dBi. The M6E nano reader consumes 0.84 W in operating mode, 15 mW in sleep mode, and 0.25 mW in shutdown mode. It is rated to read up to 200 RFID tags per second and at a reading range of 4.5 m.

**Figure 11:**
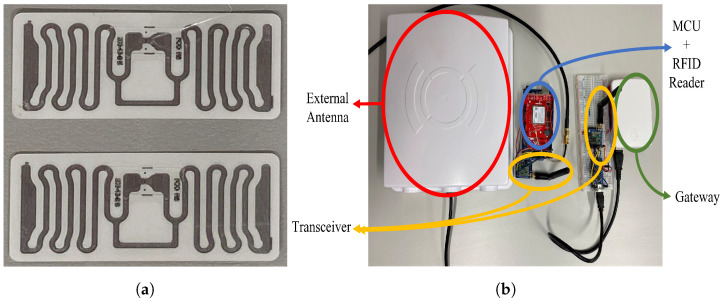
Hardware of ultra-high frequency system (**a**) RFID tags, (**b**) RFID system put together.

Tags: RFID tags are typically made up of an antenna and a microchip; the microchip provides the tag with computation and storage [34]. There are two main categories of RFID tags: passive and active. Passive tags do not have any self-energy source [38], however, when the tag comes within the range of a transmitted reader signal, its antenna is activated, creating a magnetic field and thus powering the microchip for transmitting back the tag’s unique data (a unique 32/64 bit code) to the reader. This tag has a long lifetime, but shorter reading distance. Conversely, an active RFID tag transmits its own signal to a RFID reader and requires an inbuilt battery source. These active tags have larger reading distance, but significantly higher cost. The read range of an active tag can go as far as 30 m compared to the maximum of only 6 m for a passive tag [80,81]. The RFID tags used in this study are passive due to price consideration and are shown in Figure 11a.

- HF RFID system: The RFID tag contains 1024 bits of memory and operates at the same 13.56 MHz frequency as the reader.
- UHF RFID system: The tags are adhesive based on the Electronic Product Code global (EPCglobal) Gen2 standards containing 800 bits of memory.

Transceiver: The transceiver module used in this study is the RFM69HCW. Its radio module operates in the unlicensed ISM (industry, science and medicine) band, in either 433 MHz (used in this study for better system power consumption) or 868/915 MHz. The operating voltage ranges from 3.3 V to 5 V and draws current up to 150 mA. The RFM69HCW module does not come with an inbuilt antenna, so instead an external 2.4 GHz dipole swivel antenna was soldered to the transceiver module, operating at a gain of 2 dBi. Figure 11b shows the UHF beacon and transceiver connected together. The RFM69HCW transceiver then uses an SPI to communicate with a host microcontroller (the Arduino ATmega328P on the transmitter side and the Arduino mini on the receiver side). It features advanced encryption standard (AES) encryption to keep data private from the readers to the gateway.

Gateway: The gateway is built using Raspberry Pi 4 and the RFM69HCW transceiver module for receiving information from the MCU/RFID reader. They are connected via UART serial communication as shown in Figure 11b. The prices for the hardware materials used for the HF and UHF RFID systems are shown in Table 3.

### 4.2. Software

**Figure 12:**
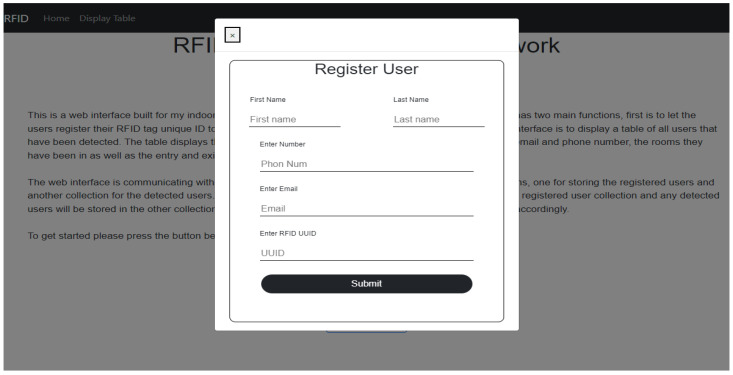
RFID system registration web interface.

Because the RFID tags hold at most 1024 bits of memory, they would not have enough memory to hold all the relevant information needed. Therefore, first the web interface registers the unique RFID tags ID through manual registration, as shown in Figure 12. It connects these tags to the users along with some contact details, storing them in a separate database called *registerUsers*. The RFID tags instead store only their unique ID. The room number, on the other hand, was pre-programmed on the MCU, as it is assumed there is always an MCU/Reader pair in each room.

**Figure 13:**
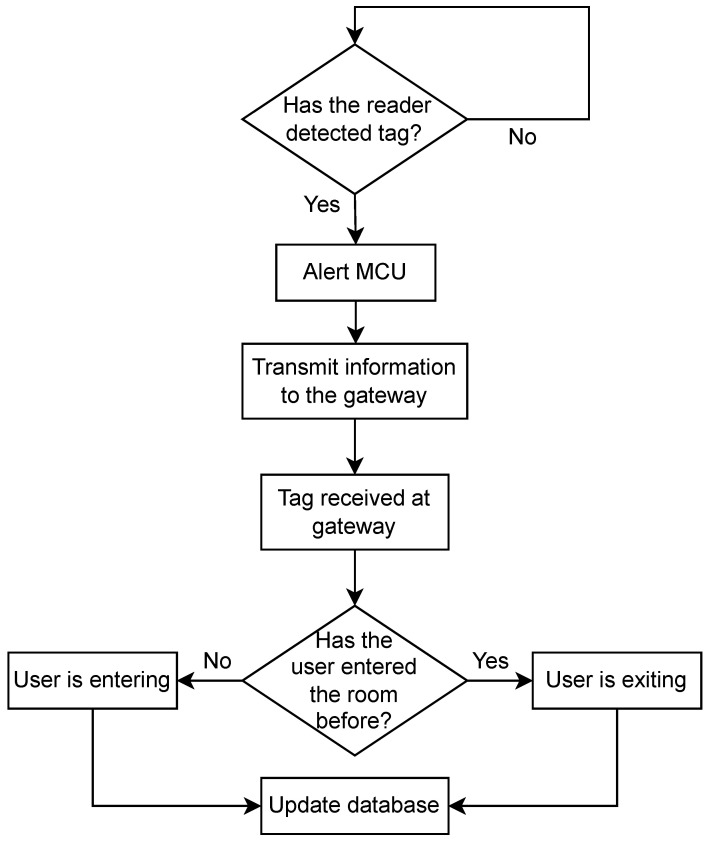
RFID algorithm 2 software overview.

Figure 13 shows the operation flowcharts for The RFID system. The readers are continuously scanning for tags; whenever a tag is identified, the MCU retrieves the data from the tags, measures the RSSI value of the back-scattered signals, concatenates it with the pre-programmed room name, and activates the transceiver to transmit these data to the gateway. Finally, the gateway extracts the relevant information from the received message, looks up the tag based on the unique RFID ID in its private *registerUsers* database, and starts inputting a detected user into the *detectedUser* database, which has the following information:

- Contact details:
  – First and last name
  – Email
  – Phone number
- The room the user has entered
- The entry and exit time of users of a particular room
- RSSI

The entry and exit tag detection process was tested by three different algorithms. Algorithm 1 is an RSSI gradient-based method: a positive gradient of the RSSI values was expected when entering (as the tag approaches the beacon), and a negative gradient was expected when leaving a room. However, it was found that the multi-path and interference distorted the RSSI values and the gradient was incorrectly recognized. Therefore, Algorithm 2 was implemented instead; the gateway handles current entry and exit detection, as shown in Figure 13.

Once the data are received at the gateway, tag detection is dependent on the tags’ past history; the gateway checks the database to see whether there is any history between the associated tag and room. If there is a history, the gateway alters the state of the tag (if the last state was entry then its new state is exit, and vice versa). Once a decision is made, the database is updated with the entry/exit time.

**Figure 14:**
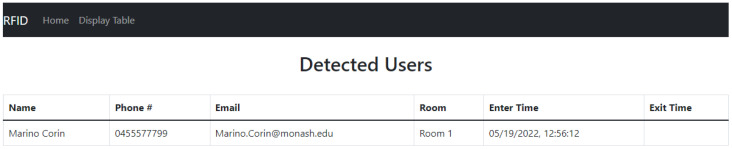
RFID system web interface displaying detected user after a tag was found.

Close contact is determined when two tags are detected for entry at the same time interval in the same room. The system first captures their RSSI entry values and compares these values. If their difference is less than or equal to 3.5 (based on practical tests of tags that were 1 m apart), the system deduces that the two tags are in close proximity. Algorithm 3 uses two beacons in the same room; it was used to overcome some flaws in Algorithm 2 that will be thoroughly explained in Section 5. In this study, a web interface front-end was developed using the MEAN stack (MongoDB ExpressJS Angular Nodejs) for easy integration with the main database that uses MongoDB and is hosted on the Raspberry Pi. The web interface front-end has two main features. The first is a user interface to allow registration for users’ details with their associated RFID unique ID as aforementioned. The second main feature a display of the *detectedUser* database in a more user-friendly table. An example of the display table is shown in Figure 14.

One potential limitation in this study is the security of the system; data encryption in the database and the addition of server level and database level permissions are among the proposed future system enhancements.

**Optimal Reader Placement:** One important parameter for the RFID system was the optimal reader placement for enhancing the scanning area, as the system depends on the LoS communication. There were two positions tested: on the side of the entrance and in front of the entrance. Each test scenario was run five times. The results, shown in Table 4, demonstrate that when the antenna is put in front of the door, the reading region is significantly wider than when it is placed to the side. The green highlighted table cells mean successful attempts, and red highlighted table cells mean unsuccessful attempts. In front of the entrance, the system had a 100% success read rate for both entering and exiting; however, when the antenna was placed on the side of the entrance, it had a 100% entry detection success but only a 40% exit detection success. The beacons placed in front of the entrance will be used throughout the upcoming tests.

## 5. Systems Performance and Efficiency Tests

**Figure 15:**
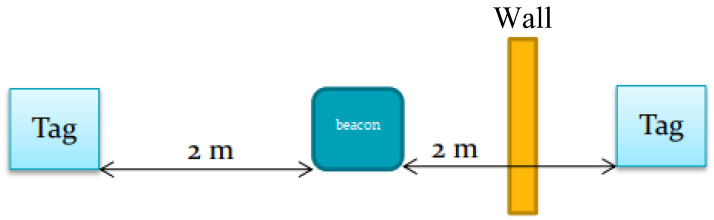
Test setup for detecting a wall effect on RSSI.

To test the effectiveness of our systems, a set of tests were done for each device. These tests are as follows:

- Effect of Walls on RSSI: To test the effect of the walls on the RSSI transmission/reception and the antenna’s bound of vision, a test was conducted according to Figure 15. A beacon was placed in a room 1 m from a 10 cm thick wall. Two tags are used, one inside the room placed 2 m from the beacon and one outside the room placed 90 cm (for a total of 2 m from the beacon) from the wall.
- Room Entrance and Exit Registration: This test was made to check the system’s ability to differentiate the entrance/exit of a tag/different tags in different rooms and precisely save the entrance and exit times.
- Identifying Close Contacts: This test was made to check the system’s ability to detect human–human proximity inside rooms between different tags for contact tracing necessities.

### 5.1. BLE Device Performance

**Figure 16:**
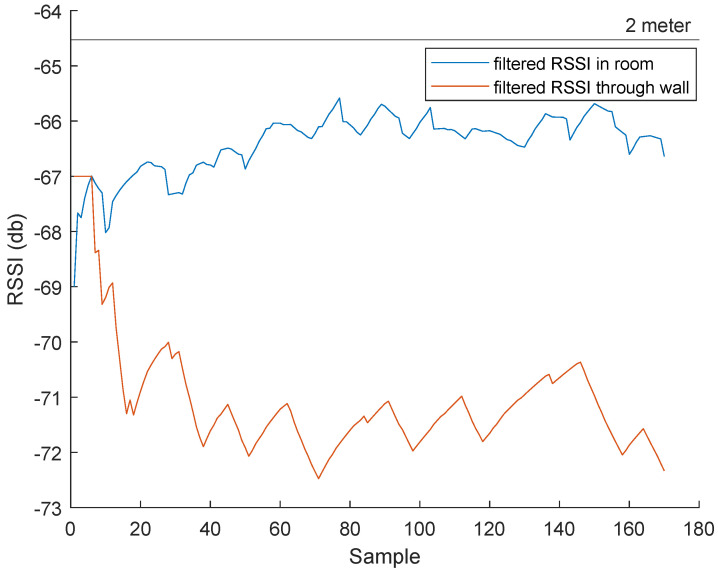
The effect of the wall on RSSI at 2 m.

**Figure 17:**
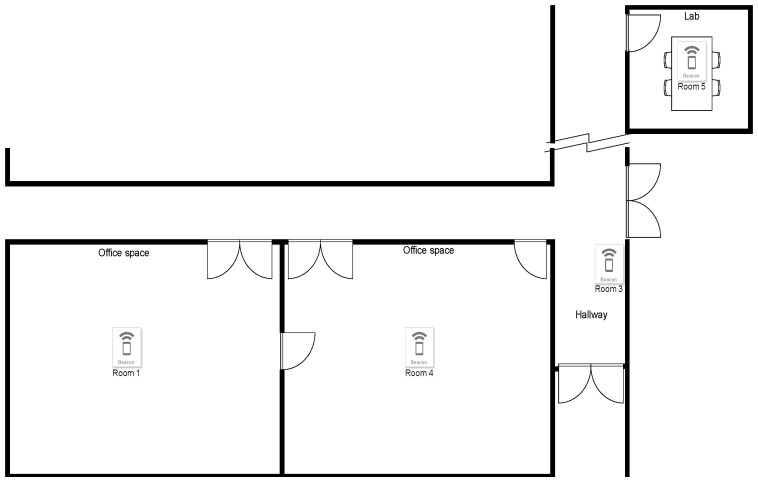
Layout of beacons in test environment at the ECSE building at Monash University.

- Effect of Walls on RSSI: From Figure 16, an average of 5 dB difference can be seen at 2 m by having a wall in the way. Although this result will change depending on the nature of the walls, this test suggests that the system can differentiate what room a tag is in while using RSSI as a localization method.
- Room Entrance and Exit Registration: To conduct this test, multiple beacons were placed inside multiple rooms at the electrical and computer system engineering (ECSE) building at Monash University, as seen in Figure 17.

**Figure 18:**
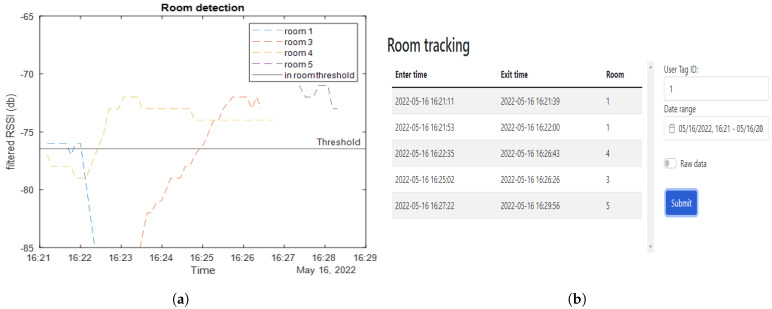
The figures show (**a**) RSSI from beacons in different rooms while moving between rooms, (**b**) web app showing where tag 1 has been within a set time frame.

This test involves a tag that starts in room 1, moves to room 4, then to room 3, and finally to room 5. As shown in Figure 18a, the received RSSI value of a specific beacon increases above the threshold value when the tag enters its room.

Some issues were noticed, such as the different effects different room architectures have on the RSSI values. For example, room 1’s beacon is very quiet even though the tag moved right next to it compared to the beacon in room 4. The wall between room 1 and 4 attenuated the signal between these rooms well, whereas the glass window between room 4 and room 3 did not. The output of these data to the end user show the entry and exit times for each of the rooms, which can be seen in Figure 18b. In the output, room one is listed twice because, as can be seen in Figure 18a, room 1’s RSSI dips below the threshold even though the tag has not left the room.

**Figure 19:**
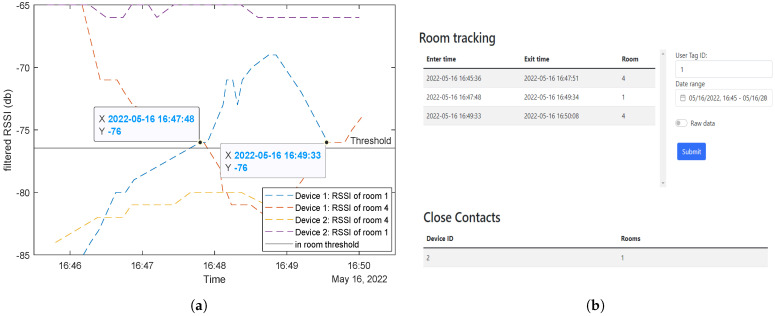
Close contact detection first test results: (**a**) RSSI values, (**b**) web app detection.

- Identifying Close Contacts: Two separate tests were conducted to test whether the contact tracing system can detect close contacts. The first was with only one tag visiting different rooms where other tags already exist; the second was with multiple tags moving around to different rooms.
  For the first test, tag 2 stays in room 1, while tag 1 starts in room 4, moves to room 1, then returns to room 4. From the results, the system can detect that both tags 1 and 2 were in room 1 from 4:47 to a 4:49 pm (Figure 19a). This matches up with the web app output specifying that tag 2 is a close contact and it happens in room 1 (Figure 19b).

For the second test, tag 1 starts in room 4, tag 2 in room 1, and tag 3 in room 5. Tag 2 will then join tag 1 in room 4, then leave to join tag 3 in room 5 and turn back room 4.

**Figure 20:**
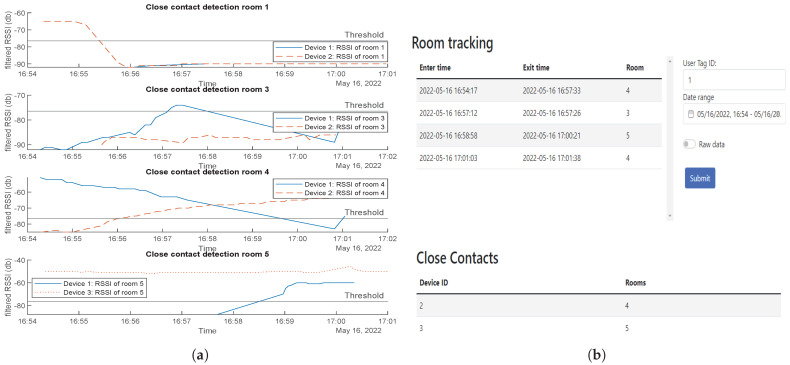
Close contact detection second test results: (**a**) RSSI values, (**b**) web app detection.

Figure 20a,b show the expected results of close contacts in RSSI values and through the web interface, respectively. One close contact happened in room 4 between tag 1 and 2 and another happened between tag 1 and 3 in room 5.

### 5.2. RFID Device Performance

Because of the short reading distance of an HF RFID system, the possible test cases were limited. The main testing case scenario for the HF RFID system was to mount the RFID reader at the entrance of a room where it allows users to tap their RFID tags. This scenario resembles the QR code check in practice, where it relies on user participation. Overall, the scenario worked as expected. However, the UHF RFID device was much more autonomous, produced more promising results, and was able to perform the contact tracing necessities much better compared to the HF system, as shown in the following tests:

- Effect of Walls on RSSI: The RFID performed the same as BLE in this test with an average drop of 5 dB difference between a tag in the same room and a tag in different room at the same distance from the reader but with a wall in between.
- Room Entrance and Exit Registration: For this test, we made multiple scenarios to test the RFID LoS effectiveness in contact tracing. The tests are listed below:
  1. First we tested three tag positions: located on a lanyard, in a pocket, and in a backpack, to get the best tag position possible for the office and hospital staff. We assessed the efficiency of the tag position by the entry and exit read rate and the accuracy of entry and exit timings. The lanyard outperformed the other two positions for having the most exposure to the antenna, as demonstrated by the findings in Table 5. The measured entry and exit times were precise, as the discrepancy between measurement and reality was only about 2 s. With the pocket and backpack having lower read rate, as shown in Table 6, the backpack placement had the lowest rate, as the tag was overly obscured, which is not recommended.
  2. Two tags; one enters; one outside: One tag will enter a room, while a second tag will wait outside. This scenario is designed to ensure that the antenna will not mistakenly detect an entry for the second tag while it is still waiting outside. As demonstrated in Table 7, the experiment was successfully replicated for all trials.
  3. Entrance to multiple rooms: For testing the system’s scalability, two antennas were placed in separate rooms to ensure that users could be tracked when accessing various rooms. The algorithm was able to locate the rooms of the tags, as indicated in Table 8.
  4. Two tags; one exits and one stays inside: In this situation, tag A will exit the room while tag B remains inside. The system mistakenly detects that tag B has kept entering and exiting the room. This is because it was in the antenna’s reading range, and the system mainly checks based on past history, assuming the user is altering his state. Results are indicated in Table 9.
  5. New entry and exit tag detection (Algorithm 3): As the last test exposed a major flaw with the current algorithm (some cases of entry and exits were mistakenly detected), a new approach was implemented. Algorithm 3 uses two antennas in each room, each with its own unique number, and the entry and exit tag algorithm searches for the sequence of these unique numbers. The unique numbers will only be 1 and 2 in this study for simplicity. The sequence for entering will be “1,2,” and the sequence for exit will be “2,1.” These unique numbers will be transmitted along with the current antenna information, and the sequence detection will be handled by the Python Gateway script in the gateway. The success of implementing this new algorithm is reflected in Table 10. The test was also rerun with several users remaining inside to assess the system’s scalability; even with multiple users inside, the system was still able to accurately detect that users did not exit (Table 11). Although there were situations when the system was unable to correctly anticipate when user A had exited, this was most likely due to the antenna positioning, which was off to the side (which was the worst performing antenna position).
- Identifying Close Contacts: The following scenario tested the entry of two tags at the same time within 1 m from each other. The system correctly detected both tags while also capturing their respective RSSI entry values, and proximity was detected from their RSSI values difference, as seen in Table 12. The reader can read up to 200 tags/s, so 1 reading every 5 ms. The RSSI values obtained in Table 12 are the results of averaging all values for the same tag occurring in 1 s.

### 5.3. BLE and RFID Together

**Figure 21:**
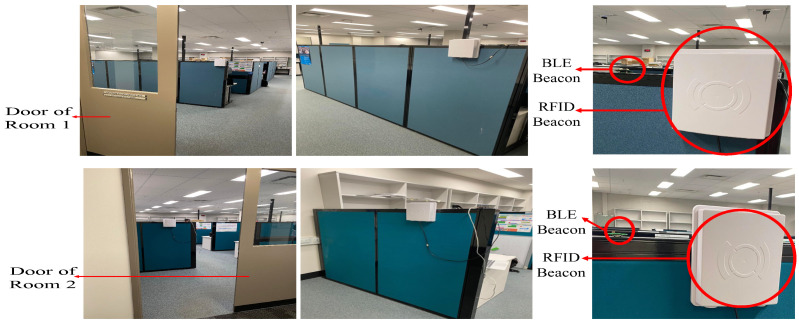
The setting for testing the two system together in Monash University. The first row shows the pictures of the first room and the second row presents the second room.

**Figure 22:**
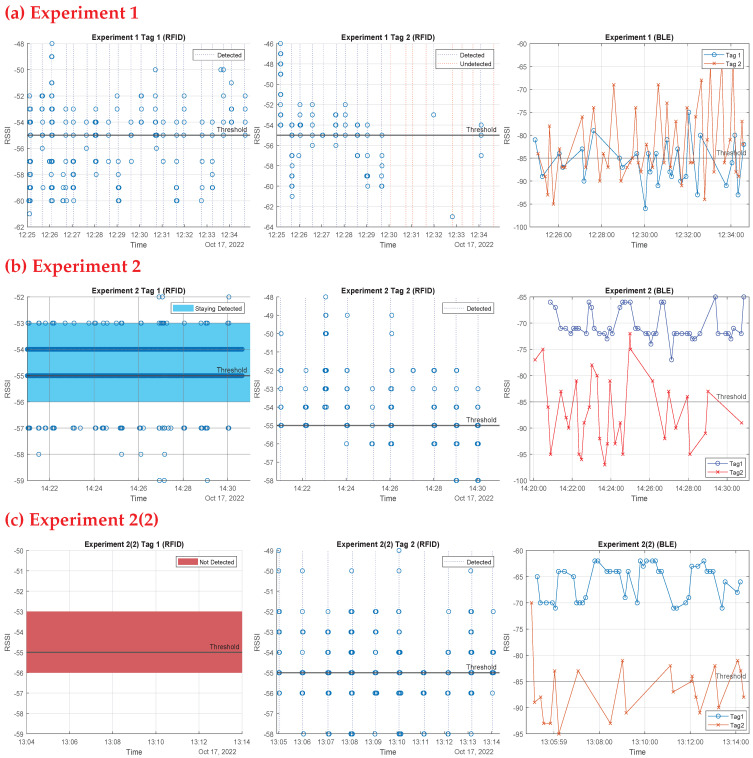
The first row shows the results of the first experiment of two tags entering and exiting together, the second row shows the results of the first trial of the second experiment of one tag exits and one tag stays, and the third row shows the results of the second trial of the second experiment. The first column represents the results of the first RFID tag, the second column represents the results of the second RFID tag, and the last column represents the results of the two BLE tags together in each experiment. In the first experiment, for RFID, the first tag is always detected (shown in blue dotted lines) while second tag has some missouts (shown in red dotted lines), the BLE system had some missouts as well. In the second experiment, for RFID, the first tag is always detected as staying (shown as blue region above threshold) while second tag’s exits were detected, also the BLE system worked fine detecting one tag always above threshold and the other tag is flactuating. In the second experiment rerun 2(2), for RFID, the first tag was not detected (shown in red region) while second tag’s exits were detected, the BLE system had the same performance as the second experiment.

**Figure 23:**
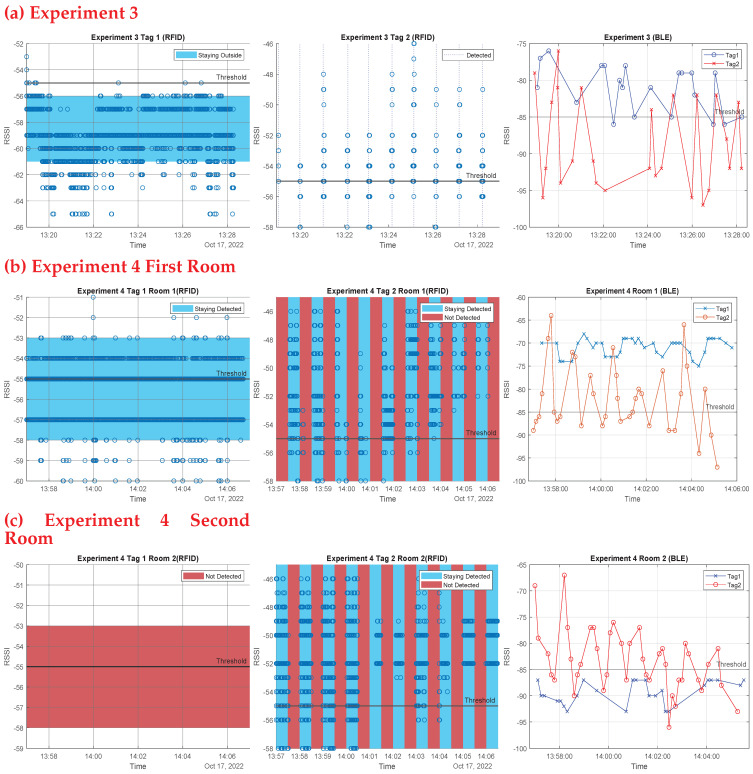
The first row shows the results of the third experiment of one tag enters and one tag outside. The second row shows the results of the forth experiment in the first room, and the third row shows the results of the forth experiment in the second room. The first column represents the results of the first RFID tag, the second column represents the results of the second RFID tag, and the last column represents the results of the two BLE tags together in each experiment. In the third experiment, for RFID, the first tag is always detected as staying outside (shown in blue region below threshold) while second tag was detected correctly, the BLE system had some miss-outs. In the fourth experiment first room, for RFID, the first tag is always detected as staying (shown in blue region above threshold) while second tag’s staying (blue regions) and leaving (red regions) durations were detected, also the BLE system worked fine. In the second room, the only difference is that the first tag was not detected (shown in red region).

**Figure 24:**
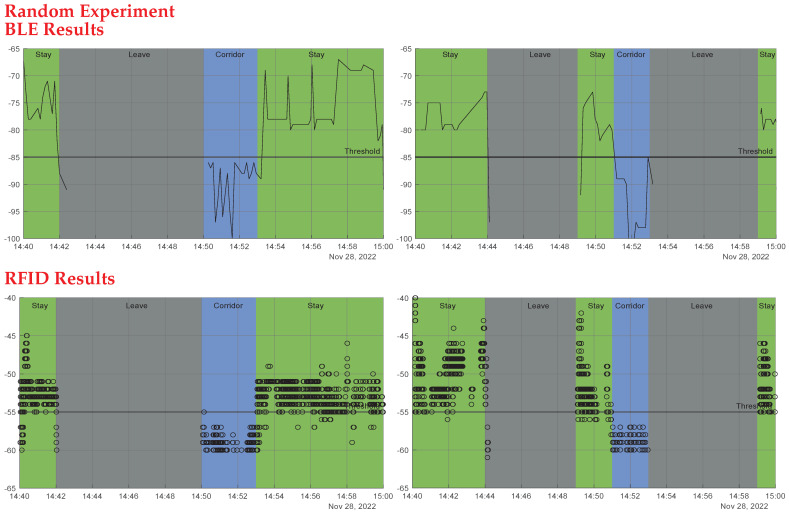
A random experiment replicating a real-life scenario in which two tagged people with tags on were required to spend their typical day in the office for 20 min. The first row shows the results of the BLE system, and the second row shows the results of the RFID system.

In a separate set of trials, we used both technologies together to test if one can compensate for the shortcomings of the other in order to reach the best approach for indoor contact tracing. This set of experiments took place in two rooms in the ECSE building at Monash University, with an RFID beacon and a BLE beacon installed in each room, as shown in Figure 21. For these experiments, the thresholding method was used in both devices (comparing the received RSSI values with predetermined threshold values for each technology to decide whether a tag is inside a room). Each person taking part in these experiments received a BLE tag as well as an RFID tag, which were pinned to their clothes. In order to thoroughly comprehend each experiment, ten attempts were made. The results of these experiments are presented as follows (experiments 1 and 2 in Figure 22, experiments 3, 4, and 5 in Figure 23):

- Experiment 1: Moving simultaneously: Two people are supposed to enter the room together then approach the beacon again while exiting after 30 s. The first person is always near the beacon while entering and exiting. This was done to test the two systems’ ability to detect two tags moving simultaneously. Figure 22 first row shows the outcomes of experiment 1: the first RFID tag was read well as the beacon sensed this tag every 30 s, whereas the other tag’s read rate was not as good, due to the blockage of the line of sight (LoS) for the second tag by the first person, who was nearer to the beacon. The success rates for RFID readings in this experiment were 100% for the first tag and 60% for the second tag. However, the BLE operated adequately, as the technology does not require a LoS. As shown in Figure 22 first row, third column, each tag was seen entering and exiting each time the RSSI was above the threshold line; the success rate for BLE readings of the first tag was 70% and for the second tag was 95%, as some entries and exits were missed due to multi-path and shadowing effects.
- Experiment 2: One person stays inside the room, while the other exits, to test the system ability to detect tags staying inside the rooms. Every two attempts were a minute apart. As seen in Figure 22 second row, the RFID system performed well. The first image depicts a continuous value above the threshold for the tag that remained in the room, whereas the second depicts the exit of the second tag at each minute. The BLE system also functioned very well in the third image, with the first tag constantly over the threshold at roughly 70 dBm and the second tag fluctuating and above the threshold at the time of the exits. RFID and BLE systems both worked flawlessly in that experiment.
  Experiment 2(2): We re-implemented this experiment; this time the person who remained in the room was out of range of the RFID beacon (behind the reader). As shown in the third row of Figure 22, the beacon missed the first tag, but detected the second tag’s departures. Again, the BLE device detected both tags, but some exits were missed.
- Experiment 3: Differentiate between inside and outside: One person is to remain just outside the room door, which has a glass window, while the other enters. This test was done to test the system sensitivity to distinguish between entry and staying outside the room. Every attempt was a minute apart from the former one. The outside tag was still seen by the beacons, but with decreased RSSI, as seen in Figure 23 first row. As a result of the door barrier, the RSSI was reduced for the RFID signal by around 4 dB and the BLE signal by about 10 dB compared to the former experiment. The other entering tag, on the other hand, was well read by both technologies. One drawback of BLE is that it detected the first tag inside the room as the RSSI measurements were above still above the threshold.
- Experiment 4: Multiple-room based experiment: Throughout the experiment, one person is intended to remain in the same room (first room). Another participant is expected to alternate between two rooms, remaining in each for 30 s. This study was carried out to assess the system’s efficiency in a multiple room-based scenario. The second row of Figure 23 shows the first room results, whereas the third row shows the second room findings.
  In the first room, the two antennas (RFID and BLE) always detected the initial tag staying in this room; for the second tag, the two antennas identified the times the second tag entered and stayed in this room. The BLE system detected a tag whose RSSI values was constantly high and above the threshold; this is the tag that stayed in the room, whereas the other fluctuating tag is the one that enters and exits, and whose RSSI rises above the threshold at the entrances and exits.
  For the second room, the RFID antenna saw only one tag at the times the person stayed in the second room. The figures also show the timing, and it can be clearly seen the periods the second tag alternated the rooms. For the BLE, one tag was constantly low and below the threshold; this is the tag staying in the other room, and this is the effect of concrete wall with no windows between rooms. Whereas the other tag was fluctuating, rising above threshold at the times of entering and exiting. Both technologies functioned decently.
- Random Experiment: Finally, we ran an experiment replicating a real-life scenario in which two tagged people were required to spend their typical day in the office for 20 minutes. We used the technologies to distinguish between three key actions: Stay (staying in the room), Corridor (leaving the room and remaining in the corridor), and Leave (leaving the room and moving away from the corridor). Figure 24 shows the results of this experiment, with the BLE system results in first row and RFID system results in second row. The Stay action needed a continuous high RSSI above the predetermined threshold value from both technologies to be identified (>−85 for BLE and >−55 for RFID), the Corridor action needed a continuous low RSSI to be identified below the predetermined threshold (<−85 for BLE and <−55 for RFID), and, finally, the Leave action needed a continuous absence of the RSSI to be identified. As seen in Figure 24, the two people’s actions were identified by the two technologies successfully and identically.

## 6. Discussion

Upon applying both technologies, some drawbacks appeared in each technology. One drawback to using the BLE is the interference with obstacles/walls, and external factors such as absorption, interference, or diffraction can affect the accuracy of calculation as RSSI tends to fluctuate. The greater the distance between the device and the beacon, the more unpredictable the RSSI becomes. The tag may mistakenly get low RSSI values and compared to the threshold that tag would be seen outside the even though the tag has not left the room. On the other hand, the RFID scanning area of the reader is limited, as it depends on LoS communications. Algorithm 2 for RFID, which depends on altering the tags states upon being detected by the antenna based on past history, showed drawbacks when a tag stayed inside the room. Consequently, Algorithm 3 was implemented that needs two readers inside each room, which can be an expensive solution. As a solution, we tested both technologies together using their RSSI values and the thresholding method. As a result we get a more affordable solution with RSSI values enhancing the performance. The BLE can get over the LoS drawback of the RFID, and, moreover, the RFID can get over the BLE RSSI fluctuation issue.

As proposed future system enhancements, we would like to increase the number of tags to practically evaluate the system’s efficiency and scalability. In addition, the system’s security and data encryption are important topics to be considered for better enhancing the systems capabilities.

## 7. Conclusions

In this research, we proposed two IoT based sensing devices for contact tracing in hospitals and offices settings, aiming to prevent personnel from cross infections, by measuring their close proximity while placing as little strain on them as possible. These devices have been demonstrated to be IoT-enabled, autonomous, low-cost, long-battery-life systems and built with off-the shelf materials for simplicity. In the first approach, a BLE device is proposed, programmed to be used in any environment, indoor or outdoor, simply by changing the path-loss exponent (n). This method locates tags by comparing their RSSI values to practically pre-determined threshold RSSI values; it can also detect close proximity between tag holders in various rooms. The second proposed method for contact tracing is based on RFID technology, and it investigates the usage of two different frequency ranges: high frequency (HF) and ultra high frequency (UHF) RFID systems (UHF). This method was successful in both indoor tag localization and detecting proximity between tags by comparing their RSSI values. Both systems were thoroughly described in terms of operation, hardware, cost, software, and web interfaces. Both systems were set up in an office/hospital-like environment and tested against various life scenarios in order to evaluate their efficiency. The results demonstrated that they met the required criteria and that they can be used to solve the previously indicated problem. It is worth noting that the approaches claimed benefits without sacrificing other aspects, such as power consumption, cost, and simplicity, which distinguish our proposed approaches from previous work. 

## Figures and Tables

**Table 1:** BLE contact tracing system hardware components and their prices.

Product/Manufacturing Process	Price (AUD)
PCB × 5 (beacon and tag paneled together)	26.13
BLE beacon components	11.79
WiFi tracing tag components	14.33
Solder stencil	9.51
Total	61.76

**Table 2:** Detailed BLE beacon advertisement packet.

Flags	Manufacturers Data	32-bit UID	TX Power	Device Name

**Table 3:** RFID contact tracing system hardware components and their prices.

System	Product	Price (AUD)
High Frequency	Arduino Uno Microcontroller	13
	RC522 RFID Reader	15.95
	Extra RFID tag/card	5.70
	Transceiver	37.22
	Total	71.87
Ultra High Frequency	ATmega2560 Arduino mega	40
	M6E Nano simultaneous RFID Reader	350.90
	RFID Tags (set of 5)	3.30
	UHF RFID Antenna (TNC)	65.50
	Interface Cable RP-SMA to U.FL	8.10
	Transceiver	37.22
	Total	505.02

**Table 4:** Repeatability test with antenna placed in front/at the side of the entrance.

Placement		Entry					Exit				
		1	2	3	4	5	1	2	3	4	5
Infront		✓	✓	✓	✓	✓	✓	✓	✓	✓	✓
Side		✓	✓	✓	✓	✓			✓		✓

**Table 5:** Lanyard placement attempts results with actual and measured timings for entrance and exit.

Lanyard	Attempt 1	Attempt 2	Attempt 3	Attempt 4	Attempt 5	Attempt 6	Attempt 7	Attempt 8	Attempt 9	Attempt 10
Enter Actual	13:50:29	13:54:19	13:56:38	14:01:02	14:10:19	14:12:28	14:16:37	14:19:10	14:20:50	14:27:44
Enter Measured	13:50:30	13:54:21	13:56:39	14:01:04	14:10:20	14:12:30	14:16:39	14:19:11	14:20:52	14:27:45
Exit Actual	13:52:10	13:55:02	13:59:19	14:09:31	14:10:40	14:14:11	14:17:26	14:19:55	14:24:02	14:29:10
Exit Measured ^1^	13:52:12	13:55:04	13:59:20	14:09:29	14:10:38	14:14:09	14:17:24	14:19:57	14:24:00	14:29:13

**Table 6:** Repeatability test with tag placed in pocket and in backpack.

Placement	Entry	Exit
Pocket	100%	90%
ine Backpack	40%	30%

**Table 7:** Two tags; one enters; one outside repeatability test results.

Attempts	1	2	3	4	5	6	7	8
First tag	✓	✓	✓	✓	✓	✓	✓	✓
Second tag	✓	✓	✓	✓	✓	✓	✓	✓

**Table 8:** Antennas in two rooms, table of results.

Attempts	1	2	3	4	5	6	7	8	9	10
Enter Room 1	✓	✓	✓	✓	✓	✓	✓	✓	✓	✓
Enter Room 2	✓	✓	✓	✓	✓	✓	✓	✓	✓	✓

**Table 9:** One user exits; one user stays inside table of results.

Attempts	1	2	3	4	5	6	7	8	9	10
Person A exits	✓	✓	✓	✓	✓	✓	✓	✓	✓	✓
Person B stays										

**Table 10:** New algorithm testing two users; one exits, and one stays, table of results.

Attempts	1	2	3	4	5	6	7	8	9	10
Person A exits	✓	✓	✓		✓	✓		✓	✓	
Person B stays	✓	✓	✓	✓	✓	✓	✓	✓	✓	✓

**Table 11:** Multiple users inside (scalability test) table of results.

Attempts	1	2	3	4	5	6	7	8	9	10
Person A exits	✓		✓	✓	✓		✓	✓	✓	
Person B stays	✓	✓	✓	✓	✓	✓	✓	✓	✓	✓
Person C stays	✓	✓	✓	✓	✓	✓	✓	✓	✓	✓
Person D stays	✓	✓	✓	✓	✓	✓	✓	✓	✓	✓
Person E stays	✓	✓	✓	✓	✓	✓	✓	✓	✓	✓

**Table 12:** Two tags entering simultaneously repeatability test.

Attempts	1	2	3	4	5	6	7	8	9	10
Person A	−54	−55	−56	−57	−54	−54	−56.5	−55.5	−57	−54.5
Person B	−55	−58	−55	−58	−55	−57	−58	−56	−56.5	−58
Difference	1	3	1	1	1	3	1.5	0.5	0.5	3.5

## Data Availability

Data available on request.

## Abbreviations

The following abbreviations are used in this manuscript:

IoT	Internet of Things
BLE	Bluetooth Low Energy
RFID	Radio Frequency Identification
HIV	Human Immunodeficiency Virus
GPS	Global Positioning System
RSSI	Receiver Signal Strength Indicator
BLE	Bluetooth Low Energy
SARS	Severe Acute Respiratory Syndrome
SoC	System on a Chip
MCU	Microcontroller
ICs	Integrated Circuits
SPI	Serial Peripheral Interface
AES	Advanced Encryption Standard
LiPo	Lithium-polymer
HF	High Frequency
UHF	Ultra High Frequency
LOS	Line of Sight
ISM	Industry, Science and Medicine Band
FSK	Frequency Shift Keying
UID	Unique Identifier
WPA 2	Wi-Fi Protected Access 2
SSID	Service Set Identifier
ECSE	Electrical and Computer System Engineering

## References

[1] Erokhin V., Gao T. (2020). Impacts of COVID-19 on trade and economic aspects of food security: Evidence from 45 developing countries. Int. J. Environ. Res. Public Health.

[2] Berasategi Sancho N., Idoiaga Mondragon N., Dosil Santamaria M., Picaza Gorrotxategi M. (2021). The well-being of children with special needs during the COVID-19 lockdown: Academic, emotional, social and physical aspects. Eur. J. Spec. Needs Educ..

[3] Stamu-O’Brien C., Carniciu S., Halvorsen E., Jafferany M. (2020). Psychological aspects of COVID-19. J. Cosmet. Dermatol..

[4] Calatrava-Nicolás F.M., Gutiérrez-Maestro E., Bautista-Salinas D., Ortiz F.J., González J.R., Vera-Repullo J.A., Jiménez-Buendía M., Méndez I., Ruiz-Esteban C., Mozos O.M. (2021). Robotic-Based Well-Being Monitoring and Coaching System for the Elderly in Their Daily Activities. Sensors.

[5] Narware A. (2020). COVID–19: Social aspects and responsibilities. Electron. Res. J. Soc. Sci. Humanit..

[6] Australian Government, Department of Health About the COVID-19 Pandemic. https://www.health.gov.au/health-alerts/covid-19/about.

[7] Kleinman R.A., Merkel C. (2020). Digital contact tracing for COVID-19. Cmaj.

[8] World Health Organization Coronavirus Disease (COVID-19): Contact Tracing. https://www.who.int/news-room/questions-and-answers/item/coronavirus-disease-covid-19-contacttracing.

[9] Shahroz M., Ahmad F., Younis M.S., Ahmad N., Boulos M.N.K., Vinuesa R., Qadir J. (2021). COVID-19 digital contact tracing applications and techniques: A review post initial deployments. Transp. Eng..

[10] Chowdhury M.J.M., Ferdous M.S., Biswas K., Chowdhury N., Muthukkumarasamy V. (2020). COVID-19 contact tracing: Challenges and future directions. IEEE Access.

[11] Gendy M.E.G., Yuce M.R. (2022). Emerging Technologies Used in Health Management and Efficiency Improvement During Different Contact Tracing Phases Against COVID-19 Pandemic. IEEE Rev. Biomed. Eng..

[12] Lai S.H.S., Tang C.Q.Y., Kurup A., Thevendran G. (2021). The experience of contact tracing in Singapore in the control of COVID-19: Highlighting the use of digital technology. Int. Orthop..

[13] Vogt F., Haire B., Selvey L., Katelaris A.L., Kaldor J. (2022). Effectiveness evaluation of digital contact tracing for COVID-19 in New South Wales, Australia. Lancet Public Health.

[14] Browne C.J., Gulbudak H., Macdonald J.C. (2022). Differential impacts of contact tracing and lockdowns on outbreak size in COVID-19 model applied to China. J. Theor. Biol..

[15] Das D.K., Khatua A., Kar T.K., Jana S. (2021). The effectiveness of contact tracing in mitigating COVID-19 outbreak: A model-based analysis in the context of India. Appl. Math. Comput..

[16] Trivedi A., Zakaria C., Balan R., Becker A., Corey G., Shenoy P. (2021). Wifitrace: Network-based contact tracing for infectious diseases using passive wifi sensing. Proc. Acm Inter. Mobile Wear. Ubiq. Technol..

[17] Reichert L., Brack S., Scheuermann B. (2021). A survey of automatic contact tracing approaches using Bluetooth Low Energy. ACM Trans. Comput. Healthc..

[18] Ekong I., Chukwu E., Chukwu M. (2020). COVID-19 mobile positioning data contact tracing and patient privacy regulations: Exploratory search of global response strategies and the use of digital tools in Nigeria. JMIR Mhealth Uhealth.

[19] Wang S., Ding S., Xiong L. (2020). A new system for surveillance and digital contact tracing for COVID-19: Spatiotemporal reporting over network and GPS. JMIR Mhealth Uhealth.

[20] Nakamoto I., Wang S., Guo Y., Zhuang W. (2020). A qr code-based contact tracing framework for sustainable containment of covid-19: Evaluation of an approach to assist the return to normal activity. JMIR Mhealth Uhealth.

[21] Luoh L. (2014). ZigBee-based intelligent indoor positioning system soft computing. Soft Comput..

[22] Roy A., Kumbhar F.H., Dhillon H.S., Saxena N., Shin S.Y., Singh S. (2020). Efficient monitoring and contact tracing for COVID-19: A smart IoT-based framework. IEEE Int. Things Mag..

[23] Kumar S., Tiwari P., Zymbler M. (2020). Internet of Things is a revolutionary approach for future technology enhancement: A review. J. Big Data.

[24] Jadon S., Choudhary A., Saini H., Dua U., Sharma N., Kaushik I. Comfy smart home using IoT. Proceedings of the International Conference on Innovative Computing & Communications (ICICC).

[25] Doshi J., Patel T., Kumar Bharti S. (2019). Smart Farming using IoT, a solution for optimally monitoring farming conditions. Procedia Comput. Sci..

[26] Agarwal V., Sharma S. (2020). IoT based smart transport management system. Proceedings of the 4th International Conference on Advanced Informatics for Computing Research.

[27] Kim T.H., Ramos C., Mohammed S. (2017). Smart city and IoT. Future Gener. Comput. Syst..

[28] Badshah A., Ghani A., Qureshi M.A., Shamshirband S. (2019). Smart security framework for educational institutions using internet of things (IoT). Comput. Mater. Contin.

[29] Datta P., Sharma B. A survey on IoT architectures, protocols, security and smart city based applications. Proceedings of the 2017 8th International Conference on Computing, Communication and Networking Technologies (ICCCNT), IEEE.

[30] Sethi P., Sarangi S.R. (2017). Internet of things: Architectures, protocols, and applications. J. Electr. Comput. Eng..

[31] Hu P., Lamontagne P. (2021). Internet of Things Based Contact Tracing Systems. Sensors.

[32] Khan M.A., Algarni F., Quasim M.T. (2020). Decentralised internet of things. Decentralised Internet of Things.

[33] Salman O., Elhajj I., Kayssi A., Chehab A. An architecture for the Internet of Tings with decentralized data and centralized control. Proceedings of the 2015 IEEE/ACS 12th International Conference of Computer Systems and Applications (AICCSA), IEEE.

[34] Bulten W., Van Rossum A.C., Haselager W.F. Human SLAM, indoor localisation of devices and users. Proceedings of the 2016 IEEE First International Conference on Internet-of-Things Design and Implementation (IoTDI), IEEE.

[35] Weis S.A. (2007). RFID (radio frequency identification): Principles and applications. System.

[36] Ngai E.W.T., Cheng T.C.E., Lai K.H., Chai P.Y.F., Choi Y.S., Sin R.K.Y. (2007). Development of an RFID-based traceability system: Experiences and lessons learned from an aircraft engineering company. Prod. Oper. Manag..

[37] Aljohani A.J., Shuja J., Alasmary W., Alashaikh A. (2021). Evaluating the dynamics of Bluetooth low energy based COVID-19 risk estimation for educational institutes. Sensors.

[38] (2016). Bluetooth SIG Proprietary Bluetooth Core Specification v5.0.XP055499587, 2822. https://www.bluetooth.com/specifications/specs/core-specification-5-0/.

[39] Kyritsis A.I., Kostopoulos P., Deriaz M., Konstantas D. A BLE-based probabilistic room-level localization method. Proceedings of the 2016 International Conference on Localization and GNSS (ICL-GNSS), IEEE.

[40] Ke C., Wu M., Chan Y., Lu K. (2018). Developing a BLE beacon-based location system using location fingerprint positioning for smart home power management. Energies.

[41] Donida B., da Costa C.A., Scherer J.N. (2021). Making the COVID-19 pandemic a driver for digital health: Brazilian strategies. JMIR Public Health Surveill..

[42] Trivedi A., Vasisht D. (2020). Digital contact tracing: Technologies, shortcomings, and the path forward. ACM SIGCOMM Comput. Commun. Rev..

[43] Foong Y.C., Green M., Zargari A., Siddique R., Tan V., Brain T., Ogden K. (2015). Mobile phones as a potential vehicle of infection in a hospital setting. J. Occup. Environ. Hyg..

[44] Cavari Y., Kaplan O., Zander A., Hazan G., Shemer-Avni Y., Borer A. (2016). Healthcare workers mobile phone usage: A potential risk for viral contamination. Surveillance pilot study. Infect. Dis..

[45] Vanany I., Shaharoun A.B.M. Barriers and critical success factors towards RFID technology adoption in South-East Asian Healthcare Industry. Proceedings of the 9th Asia Pacific Industrial Engineering & Management Systems Conference.

[46] Singapore Fights SARS with RFID. www.rfidjournal.com/article/articleview/446/1/1/.

[47] Wang S.W., Chen W.H., Ong C.S., Liu L., Chuang Y.W. RFID application in hospitals: A case study on a demonstration RFID project in a Taiwan hospital. Proceedings of the 39th Annual Hawaii International Conference on System Sciences (HICSS’06), IEEE.

[48] Athalye A., Savić V., Bolić M., Djurić P.M. A radio frequency identification system for accurate indoor localization. Proceedings of the 2011 IEEE International Conference on Acoustics, Speech and Signal Processing (ICASSP), IEEE.

[49] Mahapatra S., Kannan V., Seshadri S., Ravi V., Reka S.S. (2022). An IoT-Based Wristband for Automatic People Tracking, Contact Tracing and Geofencing for COVID-19. Sensors.

[50] Curtis S.J., Rathnayaka A., Wu F., Al Mamun A., Spiers C., Bingham G., Lau C.L., Peleg A.Y., Yuce M.R., Stewardson A.J. (2022). Feasibility of Bluetooth Low Energy wearable tags to quantify healthcare worker proximity networks and patient close contact: A pilot study. Infect. Dis. Health.

[51] Rathnayaka A., Gendy M.E.G., Wu F., Al Mamun M.A., Curtis S.J., Bingham G., Peleg A.Y., Stewardson A.J., Yuce M.R. (2023). An Autonomous IoT-based Contact Tracing Platform in a COVID-19 Patient Ward. IEEE Internet Things J..

[52] Naya F., Noma H., Ohmura R., Kogure K. Bluetooth-based indoor proximity sensing for nursing context awareness. Proceedings of the Ninth IEEE International Symposium on Wearable Computers (ISWC’05), IEEE.

[53] Rodriguez M., Pece J.P., Escudero C.J. (2005). In-building location using bluetooth. International Workshop on Wireless Ad-Hoc Networks.

[54] Zhou S., Pollard J.K. (2006). Position measurement using Bluetooth. IEEE Trans. Consum. Electron..

[55] Liu S., Jiang Y., Striegel A. (2013). Face-to-face proximity estimationusing bluetooth on smartphones. IEEE Trans. Mob. Comput..

[56] Liu S., Striegel A. Accurate extraction of face-to-face proximity using smartphones and bluetooth. Proceedings of the 20th International Conference on Computer Communications and Networks (ICCCN), IEEE.

[57] Raghavan A.N., Ananthapadmanaban H., Sivamurugan M.S., Ravindran B. Accurate mobile robot localization in indoor environments using bluetooth. Proceedings of the 2010 IEEE International Conference on Robotics and Automation, IEEE.

[58] Faragher R., Harle R. An analysis of the accuracy of bluetooth low energy for indoor positioning applications. Proceedings of the 27th International Technical Meeting of The Satellite Division of the Institute of Navigation (ION GNSS+ 2014).

[59] Faragher R., Harle R. (2015). Location fingerprinting with bluetooth low energy beacons. IEEE J. Sel. Areas Commun..

[60] Rida M.E., Liu F., Jadi Y., Algawhari A.A.A., Askourih A. Indoor location position based on bluetooth signal strength. Proceedings of the 2015 2nd International Conference on Information Science and Control Engineering, IEEE.

[61] Tripathy A.K., Mohapatra A.G., Mohanty S.P., Kougianos E., Joshi A.M., Das G. (2020). EasyBand: A wearable for safety-aware mobility during pandemic outbreak. IEEE Consum. Electron. Mag..

[62] Proximity Detection & Contact Tracing for COVID-19. Abeeway. https://www.abeeway.com/proximity-detection-contact-tracingfor-COVID-19-2/.

[63] Sattler F., Ma J., Wagner P., Neumann D., Wenzel M., Schäfer R., Samek W., Müller K.R., Wiegand T. (2020). Risk estimation of SARS-CoV-2 transmission from bluetooth low energy measurements. NPJ Digit. Med..

[64] Peng Z., Huang J., Wang H., Wang S., Chu X., Zhang X., Chen L., Huang X., Fu X., Guo Y. (2021). BU-trace: A permissionless mobile system for privacy-preserving intelligent contact tracing. Proceedings of the International Conference on Database Systems for Advanced Applications.

[65] Yu J., Chen L., Zhang R., Wang K. (2017). Finding needles in a haystack: Missing tag detection in large RFID systems. IEEE Trans. Commun..

[66] Lin K., Chen H., Ai X., Shakhov V., Ni L., Yu J., Li Y. (2020). EUMD: Efficient slot utilization based missing tag detection with unknown tags. J. Netw. Comput. Appl..

[67] Lin K., Chen H., Dai T., Liu D., Liu L., Shi L. (2018). Segmented Bloom filter based missing tag detection for large-scale RFID systems with unknown tags. IEEE Access.

[68] González-Cabañas J., Cuevas Á., Cuevas R., Maier M. (2021). Digital contact tracing: Large-scale geolocation data as an alternative to Bluetooth-based apps failure. Electronics.

[69] (2022). Design and Development Today. Judge OKs Ankle Monitors for Virus Scofflaws. https://bit.ly/3eSMZ5D.

[70] (2017). nRF52810 Product Specifications. https://infocenter.nordicsemi.com/index.jsp?topic=%2Fps_nrf52810%2Fkeyfeatures_html5.html.

[71] Wallace R. (2010). Antenna Selection Guide.

[72] Online Power Profiler for Bluetooth Le, Online Power Profiler for Bluetooth LE—opp—Online Power Profiler—Nordic DevZone. Nordic Semiconductors. https://devzone.nordicsemi.com/power/w/opp/2/online-power-profiler-for-bluetooth-le.

[73] RP515 PDF, RP515x Series Ultra-low Power Consumption 300 mA Buck DC/DC Converter with Battery Monitor. Scribd. https://www.scribd.com/document/448269327/rp515-pdf.

[74] Liu J., Chen C., Ma Y. (2012). Modeling neighbor discovery in bluetooth low energy networks. IEEE Commun. Lett..

[75] Röbesaat J., Zhang P., Abdelaal M., Theel O. (2017). An improved BLE indoor localization with Kalman-based fusion: An experimental study. Sensors.

[76] George M.M., Vadivukkarasi K. (2015). Kalman Filtering For RSSI Based Localization System in Wireless Sensor Networks. Int. J. Appl. Eng. Res..

[77] Wang J., Park J.G. (2020). A novel indoor ranging algorithm based on a received signal strength indicator and channel state information using an extended kalman filter. Appl. Sci..

[78] Guirado S.L. (2013). Localization of RFID Tags (Fixed Readers).

[79] Arduino docs: Arduino Documentation, Arduino Docs | Arduino Documentation. https://docs.arduino.cc/.

[80] Cannon J.C. (2004). Privacy: What Developers and IT Professionals Should Know.

[81] Evans N.D. (2003). Business Innovation and Disruptive Technology: Harnessing the Power of Breakthrough Technology... for Competitive Advantage.

